# A Novel Missense Mutation in *ADAMTS10* in Norwegian Elkhound Primary Glaucoma

**DOI:** 10.1371/journal.pone.0111941

**Published:** 2014-11-05

**Authors:** Saija J. Ahonen, Maria Kaukonen, Forrest D. Nussdorfer, Christine D. Harman, András M. Komáromy, Hannes Lohi

**Affiliations:** 1 Department of Veterinary Biosciences and Research Programs Unit, Molecular Neurology, University of Helsinki, Helsinki, Finland; 2 The Folkhälsan Institute of Genetics, Helsinki, Finland; 3 Department of Small Animal Clinical Sciences, College of Veterinary Medicine, Michigan State University, East Lansing, Michigan, United States of America; 4 Department of Clinical Studies, School of Veterinary Medicine, University of Pennsylvania, Philadelphia, Pennsylvania, United States of America; University of Iowa, United States of America

## Abstract

Primary glaucoma is one of the most common causes of irreversible blindness both in humans and in dogs. Glaucoma is an optic neuropathy affecting the retinal ganglion cells and optic nerve, and elevated intraocular pressure is commonly associated with the disease. Glaucoma is broadly classified into primary open angle (POAG), primary closed angle (PCAG) and primary congenital glaucoma (PCG). Human glaucomas are genetically heterogeneous and multiple loci have been identified. Glaucoma affects several dog breeds but only three loci and one gene have been implicated so far. We have investigated the genetics of primary glaucoma in the Norwegian Elkhound (NE). We established a small pedigree around the affected NEs collected from Finland, US and UK and performed a genome-wide association study with 9 cases and 8 controls to map the glaucoma gene to 750 kb region on canine chromosome 20 (p_raw_ = 4.93×10^−6^, p_genome_ = 0.025). The associated region contains a previously identified glaucoma gene, *ADAMTS10*, which was subjected to mutation screening in the coding regions. A fully segregating missense mutation (p.A387T) in exon 9 was found in 14 cases and 572 unaffected NEs (p_Fisher_ = 3.5×10^−27^) with a high carrier frequency (25.3%). The mutation interrupts a highly conserved residue in the metalloprotease domain of ADAMTS10, likely affecting its functional capacity. Our study identifies the genetic cause of primary glaucoma in NEs and enables the development of a genetic test for breeding purposes. This study establishes also a new spontaneous canine model for glaucoma research to study the *ADAMTS10* biology in optical neuropathy.

## Introduction

Glaucoma is an optic neuropathy affecting the retinal ganglion cells and optic nerve. Elevated intraocular pressure (IOP) is commonly associated with the disease. However, normal tension glaucoma is diagnosed as well [1]. Human glaucomas form a heterogeneous group of diseases, which are broadly classified into primary open-angle (POAG), primary closed-angle (PCAG) and primary congenital glaucoma (PCG) [2]. POAG is the most common form in humans [2]. In POAG, the iridocorneal angle (ICA) is open with an elevated IOP, which is considered as a significant risk factor for the disease. PCAG results from the collapse of the ICA structures, elevated IOP and subsequent death of the retinal cells [3]. Elevated IOP is caused by the blockage of the aqueous humor outflow due to a shallow anterior chamber combined with the obstruction of the iris-trabecular meshwork in the iridocorneal angle of the eye, [4]. PCG occurs within the first few years of life and is characterized by abnormalities in the anterior chamber angle and elevated IOP [4].

In human, several loci [5]–[6] and several genes including, *contactin 4* (*CNTN4*) [7], *myocilin (MYOC)* [8], *neurotrohin 4 (NTF4)* [9], *optineurin (OPTN)* [10] and *WD repeat domain 36 (WDR36)* [11] have been associated with POAG. Three loci [6] and the two genes *cytochrome P450 1B1 (CYP1B1)* [12] and *latent transforming growth factor beta binding protein 2 (LTBP2)* [13] have been associated with PCG. Three loci have been associated with PCAG, in which separate markers showed significant association after replication on human chromosomes 1, 8 and 11 [14]. Only one causative gene *ATP-binding cassette, sub-family C (CFTR/MRP), member 5* (*ABCC5*) has been associated with PCAG [15]. Furthermore, multiple genes and loci have been associated with syndromes and other ocular conditions accompanied by glaucoma [6]. However, glaucoma is considered a multifactorial disease and although several variants have been identified for familial cases, the glaucoma genetics remains controversial [16]–[17].

In dogs, POAG and PCAG have been described in several breeds [18]. Clinical features resemble human glaucoma, including loss of the retinal ganglion cells and elevated IOP. Abnormalities in the pectinate ligament (PL) structure are considered as a risk factor in canine PCAG [19]–[24]. The pectinate ligament forms the internal boundary of the canine ICA and is presented as pillar of tissue. It project from the base of iris to the peripheral Descemet’s membrane. It provides support for the iris to the posterior cornea [25]. Although structural abnormalities are considered a risk for the disease, not all dogs affected with PLD develop glaucoma, suggesting other genetic risk factors.

Despite the presence of glaucoma in many breeds the genetic etiology of glaucoma is almost completely unknown in dogs. A novel locus was mapped in Dandie Dinmont Terriers [19] and two loci were suggested for the Basset Hound [26] with PCAG. In a research colony of Beagles a recessive mutation (p.G661R) in the *ADAM metallopeptidase with thrombospondin type 1 motif*, 10 (*ADAMTS10*), has been found in a research colony of Beagles with POAG has been suggested as causative [27]. The early clinical signs in the affected Beagles include open ICA that narrows as the disease progresses, gradual increase in IOP that eventually leads to retinal ganglion cell death, optic nerve atrophy, and irreversible blindness [28]. The age of onset in Beagles varies from 8 to 16 months [29].

In addition to Beagles, Norwegian Elkhounds are also affected with POAG [30]–[31]. Unlike the Beagle, the disease in NEs is commonly diagnosed in middle-aged or elderly dogs when it starts to affect the dogś hunting capabilities [30]. However, the actual disease onset may be much earlier. Early clinical signs in NEs include a slightly elevated IOP with a normal opening of the ciliary cleft [30]. The peripheral vision is commonly affected. At the later stages of the disease, the narrowing of the ciliary cleft contributes to the elevation of IOP. This may lead to secondary subluxation of the lenses in some cases. The vision deterioration continues to expand due to optic nerve atrophy and cupping. The retina appears funduscopically normal until the late stages of the disease. Other clinical signs include Haabs striae, cataract, and buphthalmos [30].

We aimed to find the genetic cause of primary glaucoma in NEs in this study to better understand the molecular pathogenesis, to establish a large, spontaneous canine model for POAG research, and to develop a genetic test for breeding purposes. We discovered a novel missense mutation in the *ADAMTS10* gene.

## Materials and Methods

### Study cohort

Blood samples from 596 NEs, including 16 glaucoma affected dogs from Finland, Norway, Sweden, United Kingdom and United States were collected to the canine DNA bank at the University of Helsinki, Finland with owneŕs consent and under the permission of the Animal Ethical Committee of County Administrative Board of Southern Finland (ESAVI/6054/04.10.03/2012). All affected dogs were examined by a certified veterinary ophthalmologist. The clinical diagnoses indicated bilateral primary glaucoma with significantly elevated IOP, between 25–86 mmHg (normal 10–20 mmHg), and no detectable underlying cause. In addition, various degrees of optic nerve atrophy and cupping were reported. In addition, a progressive vision loss was detected in the affected dogs leading to complete blindness. Other, secondary clinical signs included lens luxation, corneal stromal edema, Haabs striae, keratitis, vitreal syneresis and retinal degeneration. The average age at the time of diagnosis was 6.5 years.

Genomic DNA was extracted from EDTA blood using Chemagic Magnetic Separation Module I (MSM I) (Chemagen Biopolymer-Technologie AG, Baeswieler, Germany) according to the manufacturer’s instructions. DNA from buccal swabs (Eurotubo Cytobrush, sterile, 200 mm, Danlab, Helsinki, Finland) was extracted using QIAamp DNA Mini Kit (Qiagen).

### Genome wide association study

**Figure 1:**
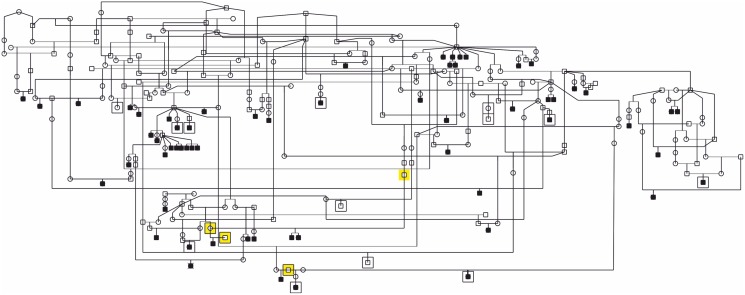
Pedigree of glaucoma affected Norwegian Elkhounds. The pedigree constructed around affected dogs indicates a likely recessive mode of inheritance as the affected dogs are born to unaffected parents and there are multiple affected littermates in some litter. The squared dogs were included in the GWAS. Individuals marked with yellow background were genotyped as obligatory carriers and were all heterozygous for the mutation.

**Figure 2:**
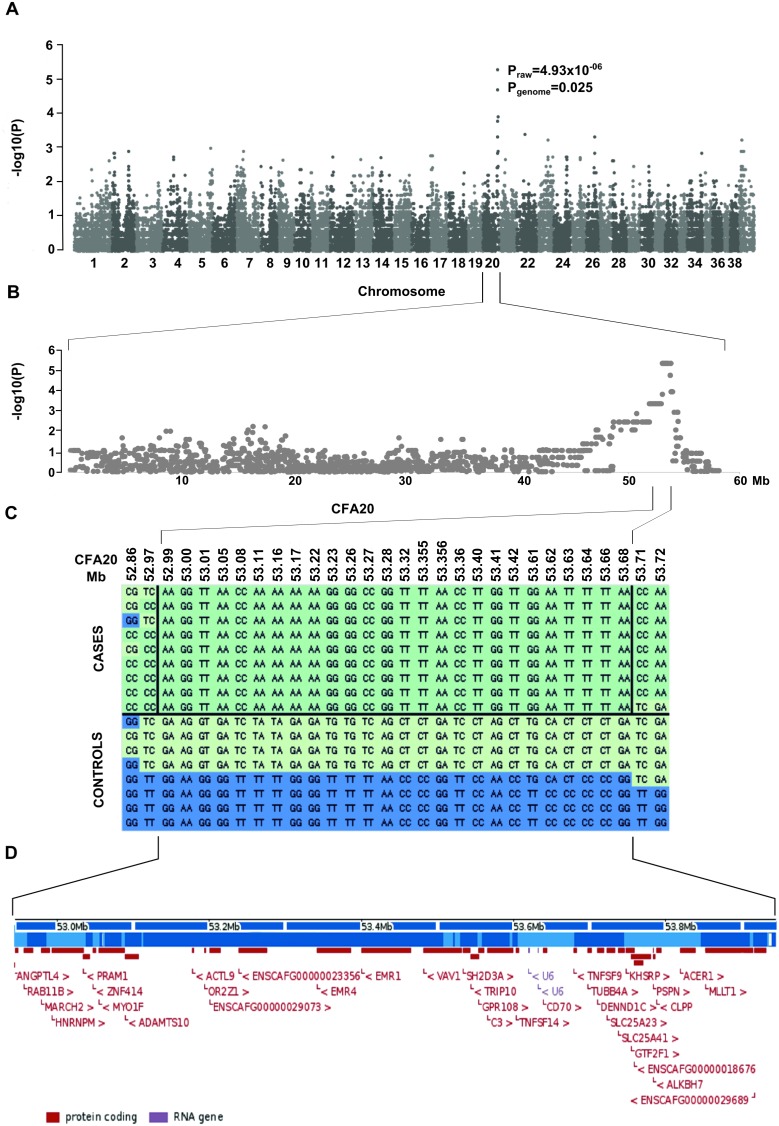
Genome wide association study. **A**) A Manhattan plot of genome-wide case-control association analysis with 8 cases and 9 controls indicate the most highly associated region in CFA20. **B**) The glaucoma associated region on chromosome 20 spans from 53.1 Mb to 53.8 Mb. **C**) Genotypes at the associated region on CFA20. All cases share a 750 kb homozygous block. **D**) The associated region harbors 35 genes of which only *ADAMTS10* has been associated with POAG.

A genome-wide association study (GWAS) was performed using Illumina’s CanineHD BeadChip array (San Diego, CA, USA) with 9 cases and 8 controls (**Fig. 1**). The control dogs were at least 8 years of age without any clinical signs of glaucoma. Genotyping was performed at the Geneseek (Lincoln, NE, USA) and the genotyping data was analyzed using PLINK 1.07 analysis software. A total of 173,662 markers were initially included for the analysis. No individual were removed for low genotyping success of 95%. Missingness test of 95% removed 17,484 SNPs. A total of 72,715 SNPs had minor allele frequency of less than 5% and were removed. None of the SNPs deviated from Hardy-Weinberg equilibrium based of HWE test of P< = 0.0001. After frequency and genotyping pruning, 89,277 SNPs remained in the analysis. A case-controls association test was performed using PLINK software to compare the allele frequencies between cases and controls (**Fig. 2A**). Identity-by-state (IBS) clustering and CMH meta-analysis (PLINK) were used to adjust for population stratification. Genome-wide corrected empirical p-values were determined applying 100,000 permutations to the data. Besides PLINK the data was analyzed with compressed mixed linear model [32] implemented in the GAPIT R package [33] and with R-implemented GenABEL [34] software (data not shown). The GWAS data is publicly available at dbSNP database (http://www.ncbi.nlm.nih.gov/projects/SNP/).

### Candidate gene sequencing

The coding regions of the best candidate gene in the associated region, *ADAMTS10,* were first sequenced in four NE cases, in four NE controls (unaffected >8 years) and in one unaffected Rough Collie samples. The identified candidate mutation was then validated in 596 NEs, including 7 additional cases. In addition, the mutation was studied in 71 dogs from 17 other breeds affected with POAG, PCAG or PLD and in 115 unaffected dogs from 6 breeds (**Table S1**).

Primer pairs (**Table S2**) were designed for the ADAMTS10 gene to amplify the coding regions and splice sites by standard PCR (**Table S2**). PCRs were carried out in 12 µl reactions consisting of 1.2 U Biotools DNA Polymerase (Biotools, Madrid, Spain), 1.5 mM MgCl_2_ (Biotools, Madrid, Spain), 200 µM dNTPs (Finnzymes, Espoo, Finland), 1 x Biotools PCR buffer without MgCl_2_ (Biotools, Madrid, Spain), 0.83 µM forward and reverse primer (Sigma Aldrich, St. Louis, USA) and 10 ng template genomic DNA. Reaction mixtures were subjected to a thermal cycling program of 95°C for 10 min, followed by 35 cycles of 95°C for 30 s, 30 s at the annealing temperature and 72°C for 60 s and a final elongation stage of 72°C for 10 min. ExoSap purified PCR fragments were Sanger sequenced in our core facility the FIMM Technology Center using ABI 3730xl DNA analyzer (Applied Biosystems, Foster City, California, USA). Sequence data analysis was performed using the Sequencher software (Gene Codes, Ann Arbor, MI, USA). Build 3.1 of the canine genome reference sequence was applied in the study.

## Results

We established a global sample cohort including glaucoma affected (n = 16) and unaffected NEs (>570) to map the disease locus, to identify the causative gene and to validate the segregation of the mutation. We performed a GWAS in a small pedigree of 9 cases and 8 controls. Statistical analyses revealed a 750 kb locus on CFA20 with 23 most highly associated SNPs between 53070684 bp to 53816416 bp (p_raw_ = 4.93×10^−06^, p_genome_ = 0.025) (**Fig. 2B, C**). A mild population stratification was identified in the study cohort by genome wide IBS clustering (genomic inflation factor λ = 1.1) (**Fig. S2**), but it did not affect the result as two mixed model approaches (GenABEL and GAPIT) that better control for population stratification, gave the same results (data not shown).

A pedigree constructed around the affected dogs using GenoPro genealogy software (http://www.genopro.com) suggested a recessive mode of inheritance as the affected dogs are born to unaffected parents and there are multiple affected littermates in some litters.

**Figure 3:**
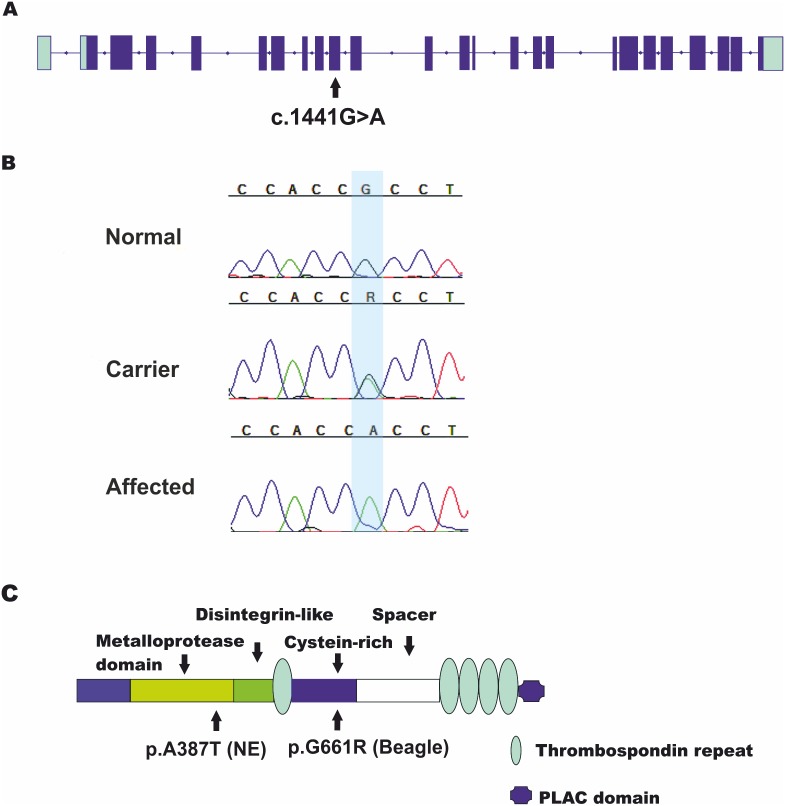
A missense mutation in the *ADAMTS10*. **A**) A schematic representation of the *ADAMTS10* gene structure. The gene is composed of 24 coding exons (dark blue) and the c.1441G>A variant is positioned in the exon 9 (not in scale). **B**) Chromatograms of the non-synonymous variant position in an affected, a carrier and a wild-type dog. **C**) The p.A387T variant is positioned in the catalytic metalloprotease domain.

The identified locus contains 35 genes including a known canine POAG gene, *ADAMTS10* (**Fig. 2D**). It was selected for mutation screening in the coding regions and splice sites in four affected and five control dogs. Sequencing identified altogether ten variants; a non-synonymous variant c.1441G>A, p.A387T in the exon 9 (**Fig. 3A, B**), five synonymous and four non-coding variants (**Table S3**). Only one out of the five synonymous variants, p.P171P, co-segregated with the non-synonymous variant. However, it was not suspected to be in the exonic enhancer region when analyzed by the ESE-finder [35]. Instead, the non-synonymous variant was predicted to be pathogenic based on the bioinformatics prediction tools Polyphen2 [36] and SIFT [37]. The mutation affects a highly conserved residue (present in 75 species, **Fig. S1**) in the metalloprotease domain of ADAMTS10 protein (**Fig. 3C**).

To gather further evidence for the p.A387T segregation, we genotyped seven additional NE primary glaucoma cases and four obligatory carriers (**Fig. 1**) and 572 randomly selected unaffected NEs. All but two of the cases were homozygous for the mutation. Unfortunately, we have a limited access to the health records and history of these two affected dogs and they may have had a secondary glaucoma with a primary underlying cause such as inflammation being missed at the end stage of disease when the animals were presented to the veterinary ophthalmologist. All four obligatory carriers were heterozygous. Genotyping of the 572 NEs indicated a 25.3% carrier frequency (151/596) and revealed one additional homozygous dog. This genetically affected dog is now 5 years old without signs of glaucoma yet but needs to be followed up since the average age of diagnosis in the breed is at 6.5 years of age. Collectively, these results support a segregation of a mutation in clinically confirmed primary glaucoma cases with a highly significant association between the mutation and disease when comparing genotyped genetically affected dogs (n = 16) and unaffected using Fisher’s exact test (P_Fisher_ = 3.5×10^−27^). The breed-specificity of the p.A387T mutation was indicated by excluding it from 71 glaucoma or PLD affected dogs from 17 breeds and from 115 unaffected dogs from six breeds.

## Discussion

Our study has identified a novel recessive mutation in the *ADAMT10* gene in the NEs affected with primary glaucoma using genome wide association analysis and candidate sequencing strategies. We mapped the disease to a known canine POAG locus including the *ADAMTS10* candidate gene and subsequently identified a missense mutation in the exon 9 of the *ADAMTS10* gene. This is consistent with the previously reported POAG phenotype of primary glaucoma in this breed [30]–[31]. The recessively segregating mutation results in an alanine to threonine change (p.A387T) in a highly conserved functional metalloprotease domain of the protein (**Fig. 3C**), which likely impairs ADAMTS10 function, leading to POAG in the homozygous dogs.

ADAMTS10 is a secreted glycoprotein [38] and belongs to a family of metalloproteinases that contribute to the dynamics of the extracellular matrix (ECM) composition and microfibril function [38]–[40]. It may have a role in the storage and activation of latent transforming growth factor beta (TGF_β_), which regulates the collagen turnover [41]–[43] as well as in the remodeling of the mesenchymal and basement membranes [44].

The members of the ADAMTS family share the same structural organization including catalytic metalloprotease domain, followed by a disintegrin-like, and cysteine-rich domains, a thrombospondin repeat and a spacer region (**Fig. 3C**). ADAMTS10 differs from the others having five thrombospondin type 1 repeats and a PLAC domain in the C-terminal [44].

ADAMTS10 interacts with fibrillin-1 and localized to fibrillin-1 microfibril bundles [39]. It maintains the lens in its position via lens ligaments, which are comprised primarily from fibrillin-1 [43]. Microfibrils localize and activate TGF_β_ [45]. Fibrillin-1 is expressed in the outflow pathway of the aqueous humor and defect in the fibrillin-1 may lead to impaired aqueous humor flow [46]–[48].

In dogs, a missense mutation (p.G661R) in the *ADAMTS10* gene has been previously associated with POAG in a research colony of Beagles. The Beagle mutation is positioned in the cysteine rich domain and is hypothesized to disrupt protein folding leading to instability [27].

The NE mutation is different from the Beagles and may result in a different pathogenesis. NE mutation changes a highly conserved residue in the metalloprotease domain, which plays a role in the remodeling of the connective tissue (**Fig. 3C**) [44]. Human metalloprotease domain mutations have revealed abnormalities in the cellular cytoskeleton [44], suggesting abnormal interactions with the ECM. These abnormalities may eventually result in defective microfibrils and glaucoma through alterations in biomechanical properties of tissue and/or through effects on signaling through TGF_β_, which is known to be elevated in the aqueous humor of glaucoma patients [49]. Unfortunately, we did not have access to any tissue samples from the affected dogs to further investigate the functional consequences of the mutation for *ADAMTS10* and its pathway. Secondary lens luxation diagnosed in the affected NEs may be due to abnormal fibrillin-1 functions as the ADAMTS10 and fibrillin-1 interaction may be impaired causing disruption of the lens ligaments.

Dagoneau et al. identified three causative mutations in the metalloprotease domain of *ADAMTS10* in human WMS patients [44]. When studying patient fibroblasts they noted that abnormally large bundles of actin were present, which were reflection of cytoskeleton abnormalities as a result of impaired connections between cytoskeleton and ECM [44].

Mutations in *ADAMTS10* [44], [50], *ADAMTS17* [50] and in *fibrillin 1* (*FBN1*) [51] have been associated with a Weill-Marchesani syndrome (WMS) (OMIM 277600). WMS is a connective tissue disorder and characterized by several eye defects including glaucoma, myopia, ectopia lentis, microspherophakia and other features such as short stature, brachydactyly, joint stiffness in the hands, restricted articular movements and some facial features [52]. Glaucoma is diagnosed in WMS patients, but ectopia lentis is the more prevalent clinical sign [53]. In WMS patients the ectopia lentis and dysgenesis of the lens ligament is suspected to be caused by abnormal biogenesis of fibrillin-1 [39]. The same is likely true for *ADAMTS17*-mutant dogs [54] and *ADAMTS10*-mutant Beagles [27]. Interestingly, none of our affected NE dogs presented with signs of primary lens luxation, although this should be expected. Histologic analyses of the lens zonules would be very helpful to identify possible signs of zonular dysplasia. Another difference in the affected NE dogs relates to the lack of severe non-ocular signs, which are commonly present in the WMS patients with mutation in the *ADAMTS10* and *ADAMTS17* genes. Further studies are warranted to investigate whether the observed breed- and species–specific differences are due to alternate mutations in the metalloproteinase domains or possible other factors.

In summary, we have discovered the genetic cause of primary glaucoma in the NEs by identifying a missense mutation in *ADAMTS10*. This study implicates the significant role of ADAMTS10 in canine POAG by identifying the second mutation in the same gene in dogs. Our affected dogs establish a new model to study *ADAMTS10* biology in the microfibrillin theory of the glaucoma [49]. Importantly, given the high frequency of the mutation in the NE breed, our study is a breakthrough for the NE breeders, who will benefit from the genetic test to reduce the disease frequency in future populations.

## Supporting Information

Figure S1 **ADAMTS10 protein alignments.** ADAMTS10 sequence alignment between different species. The mutation is located in a highly conserved region across 75 species. The arrow marks the mutated alanine residue.
(ZIP)

Figure S2 **Q-Q plots.** Identity-by-state (IBS) clustering and CMH meta-analysis (PLINK) were used to adjust for population stratification (A). A mild population stratification was identified in the study cohort by genome wide IBS clustering (adjusted genomic inflation factor λ = 1.1) (B).
(TIF)

Table S1 **Dogs affected with glaucoma of PLD (71 dogs in 17 breeds) and healthy dog (115 dogs in six breeds) were sequenced for the** ***ADAMTS10*** **mutation.**
(XLSX)

Table S2 **Primers used for PCR amplification and sequencing of canine ADAMTS10 coding regions.**
(XLSX)

Table S3 **A total of 10 variants were in the coding and splice site regions of** ***ADAMTS10*** **.**
(XLSX)
