# Correction: A Novel Missense Mutation in ADAMTS10 in Norwegian Elkhound Primary Glaucoma

**DOI:** 10.1371/journal.pone.0118256

**Published:** 2015-02-17

**Authors:** The PLOS ONE Staff

Fig. 1 is incorrect. Please view a correct version here.

**Fig 1:**
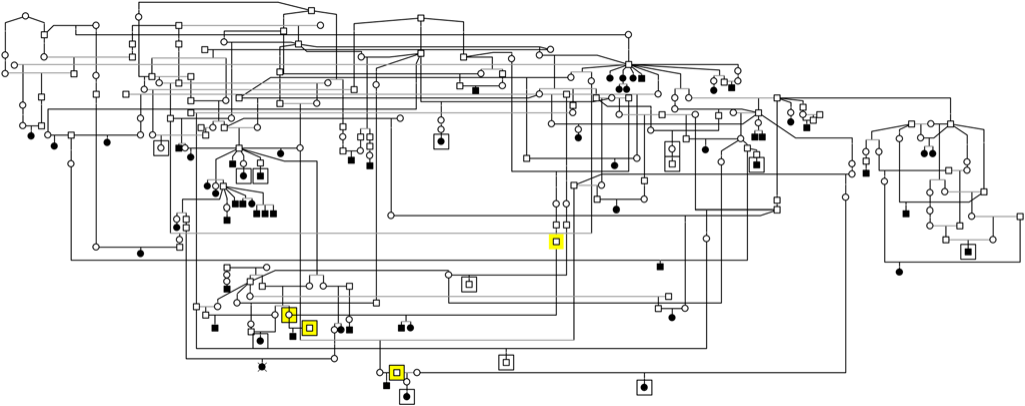
Pedigree of glaucoma affected Norwegian Elkhounds. The pedigree constructed around affected dogs indicates a likely recessive mode of inheritance as the affected dogs are born to unaffected parents and there are multiple affected littermates in some litter. The squared dogs were included in the GWAS. Individuals marked with yellow background were genotyped as obligatory carriers and were all heterozygous for the mutation.
