# Supplementary figures and images for: A Novel Missense Mutation in ADAMTS10 in Norwegian Elkhound Primary Glaucoma
Source: PLoS One. 2014 Nov 5;9(11):e111941. doi: 10.1371/journal.pone.0111941 (PMC4221187; doi:10.1371/journal.pone.0111941)

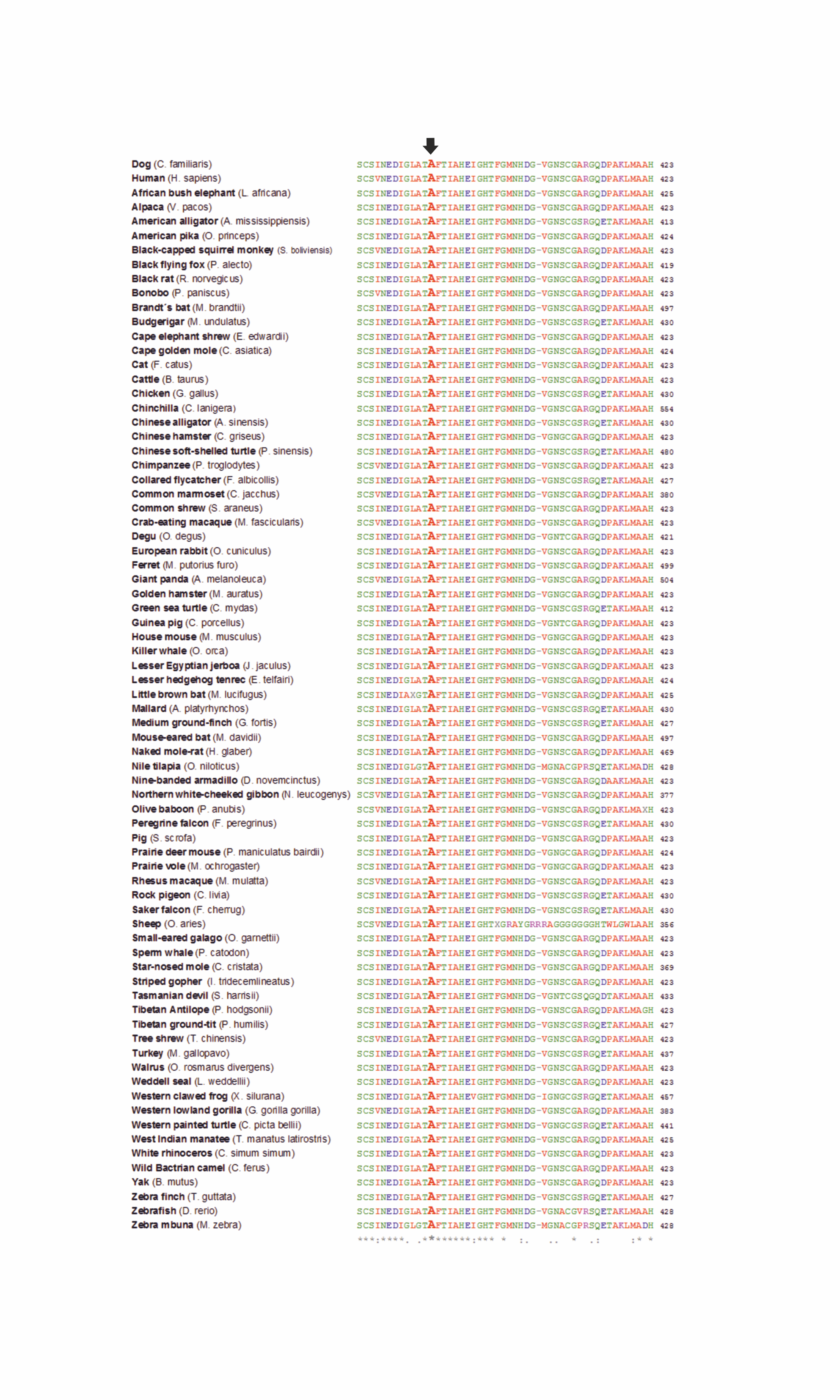

Supplement: Figure S1 — ADAMTS10 protein alignments. ADAMTS10 sequence alignment between different species. The mutation is located in a highly conserved region across 75 species. The arrow marks the mutated alanine residue. (ZIP) [file pone.0111941.s001.zip › ADAMTS10_alignment2_RBG.tif]

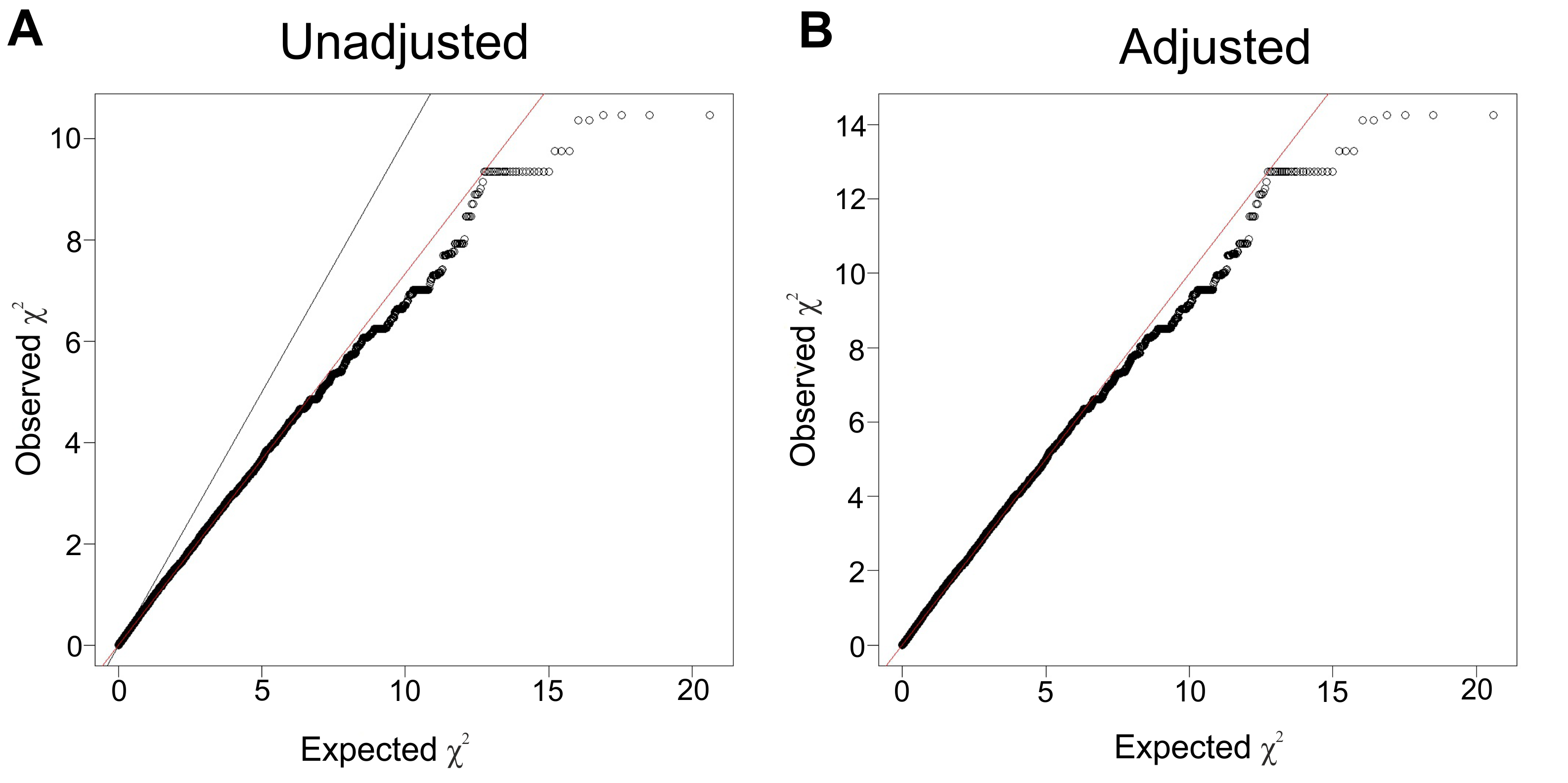

Supplement: Figure S2 — Q-Q plots. Identity-by-state (IBS) clustering and CMH meta-analysis (PLINK) were used to adjust for population stratification (A). A mild population stratification was identified in the study cohort by genome wide IBS clustering (adjusted genomic inflation factor λ = 1.1) (B). (TIF) [file pone.0111941.s002.tif]
